# *λ*/20 surface nanostructuring of ZnO by mask-less ultrafast laser processing

**DOI:** 10.1515/nanoph-2022-0657

**Published:** 2023-01-16

**Authors:** Shi Bai, Zhaoxu Li, Kotaro Obata, Shota Kawabata, Koji Sugioka

**Affiliations:** Advanced Laser Processing Research Team, RIKEN Center for Advanced Photonics, 2-1 Hirosawa, Wako, Saitama 351-0198, Japan; Hebei Key Laboratory of Materials Near-Net-Forming Technology, School of Material Science and Engineering, Hebei University of Science and Technology, Shijiazhuang 050018, China

**Keywords:** laser processing, LIPSS, plasmonic, SERS, surface nanostructuring, ultrafast laser

## Abstract

Fabrication of nanostructures with a feature size much smaller than the laser wavelength is challenging due to the optical diffraction limit. It’s well known that the irradiation of polarized ultrafast laser generates periodic nanostructures, so called laser-induced periodic surface structures (LIPSS). Owing to the modulated field, the surface is periodically ablated to form specific patterns, which can be used for some photonic applications including surface-enhanced Raman scattering (SERS). In this paper, we investigate the morphologies of LIPSS on ZnO substrates by mask-less ultrafast laser processing. By adjusting the laser processing parameters, including fluence, pulse number, polarization, and pulse duration, the homogenous nanostrip array and nanopillar array are created. Furthermore, by adjusting the laser fluence, a single nanogroove with a width of ∼20 nm and a single nanocavity with a diameter of ∼24 nm are created. The gold nanoparticles are then coated on the ZnO nanopillar array for SERS application. We found that the concentration of defects in ZnO substrate is increased by the laser irradiation, which is beneficial for SERS performances to achieve an enhancement factor of SERS as high as 2.28 × 10^7^.

## 1 Introduction

Laser-induced periodic surface structures (LIPSS), which have been found in the last century, are widely being explored in recent years to achieve periodic structures in a large area. The LIPSS with different morphologies formed on surfaces of metals, dielectrics, and semiconductors by nanosecond, picosecond (ps), and femtosecond (fs) laser have been extensively investigated [1–3]. It has been found the morphology of LIPSS is highly related to fluence, laser wavelength, pulse number, and beam polarization. These parameters are critical for the generation of homogenous LIPSS. The LIPSS can be simply classified into two types in terms of period of created structures. The low spatial frequency LIPSS (LSFL) has a period larger than half the laser wavelength (*λ*), while LIPSS with the period smaller than *λ/2* is termed high spatial frequency LIPSS (HSFL) [3]. The period of LSFL and HSFL typically depends on the types of materials in terms of absorption property [4]: for the opaque materials, a period of ∼*λ* for LSFL and ∼*λ/*2 for HSFL (LIPSS-1), while for the transparent materials, a period of ∼*λ*/*n* for LSFL (*n* is refractive index) and ∼*λ*/2*n* for HSFL (LIPSS-2). The HSFL is usually formed at low fluence and it transits to LSFL by increasing fluence and decreasing pulse numbers [5]. Currently, 1 dimensional (1D) and 2D LIPSS patterns with the dot, strip, triangle, and hexagonal shapes have been successfully created by adjusting laser polarization and pulse duration, or by using multi beams [3, 6], [7], [8].

The formation mechanism of LSFL is attributed to interference of the incident laser beam and the laser beam scattered at the surface, giving rise to periodic ablation to form the periodic structure with a period in the range of ∼*λ* [3]. The formation mechanism of HSFL is more complex and still in debate. One of the most possible mechanisms is the interference of incident laser beam and surface plasmon polaritons (SPP) generated by the incident laser beam, accompanied by self-organization of materials [4]. SPP is easily launched and supported on metallic surface. Meanwhile for semiconductor and dielectric surfaces, such as ZnO, generation of SPP was also investigated in previous studies, reporting that the fs laser-excited surfaces showed metallic properties [9, 10]. This “metal transition” is due to the high intensity of fs laser to locally generate high-density free carriers by nonlinear interaction. The free carrier density in ZnO can easily exceed 10^22^ cm^−3^, leading to the real part of permittivity smaller than −1 so as to be a metal-like surface [11]. If the HSFL is governed by the interference, the fluence of a Gaussian laser beam can be adjusted to create single or controlled number of grooves with a feature size far beyond the optical diffraction limit on the material surface [12]. For example, a recent study showed that the irradiation of double beams of 800 nm fs laser with orthogonal polarizations created a 12 nm (FWHM) groove on silicon surface [13]. However, there’s few research on the creation of a single nanocavity beyond the optical diffraction limit on material surfaces by mask-less laser processing.

In this paper, we illustrate a simple and direct approach to fabricate a *λ*/20 single nanostructure on ZnO substrate with mask-less ultrafast laser processing. We investigate fabrication of homogenous HSFL by optimizing processing parameters. Specifically, the HSFL morphologies formed by linearly polarized and elliptically polarized beams are compared. Then, we demonstrate formation of a single nanogroove, and multi grooves whose number is controlled, by linearly polarized laser beam scanning, while nanocavities with a diameter of ∼ 25 nm (*λ*/20) are generated by the circularly polarized laser beam canning. The mechanism is also discussed to understand the structure formation. We further perform the fabrication of specific patterns, including a string and a 2D array of nanocavities and nano “QR code” to show flexibility and versatility of this technique. Furthermore, the homogenous HSFL is coated by gold nanoparticles for the application to surface-enhanced Raman scattering (SERS) analysis.

## 2 Materials and methods

### 2.1 Femtosecond laser processing

The ultrafast laser processing setup is shown in Figure S1. The laser beams (wavelength of 1030 nm. CB5-06, CARBIDE, Light Conversion) with variable pulse duration and repetition rate (Rep) was employed for laser processing of polished ZnO substrate (C(0001), CrystalBase). The laser power was adjusted by the attenuator equipped on the optical path. To convert the wavelength to second harmonics (515 nm), a lithium tetraborate (LBO) crystal was used. The laser polarization was controlled by half/quarter waveplates. The objective lens (Mitutoyo) with different numerical apertures (NA) was used to focus the laser beam on the ZnO substrate. The diameter of focused spot size (*d*) was estimated by *d* = 1.22*λ*/NA, where *λ* was the laser wavelength. The average pulse fluence (*F*_*p*_) was calculated by *F*_*p*_ = 4*E*/(*πd* ^2^), where *E* was the single pulse energy. The pulse to pulse overlap (*pp*) in laser scanning was given by *pp =* (2*d*/*v*) × Rep, where *v* is the scanning speed. The accumulated *F* (*F*_*a*_) was defined as *F*_*a*_ = *F*_*p*_ × *pp.*

### 2.2 Raman measurements

Before Raman measurements, the laser nanostructured ZnO substrate was coated by gold nanoparticles using an ion coater (ESC-101SC, Elionix). The coating rate was 15 nm/min. The Raman spectra were recorded by a Raman spectrometer (NRS-4500, JASCO) equipped with a 633 nm excitation laser. The grating in the spectrometer had 1800 grooves/mm and the resolution of Raman shift was 2.3 cm^−1^. The excitation laser was focused by an objective lens with an NA of 0.5 at laser power of 5.6 mW. The exposure time was 20 s with 2 accumulations.

### 2.3 Characterization

The morphology and the energy-dispersive X-ray spectroscopy (EDS) of samples were analyzed using a field emission scanning electron microscopy (SEM) (Quattro, Thermo Fisher Scientific). The nanostructured ZnO was characterized using X-ray diffraction (XRD) at room temperature (Rigaku SmartLab).

## 3 Results and discussion

### 3.1 Homogenous HFSL

To form HFSL, *F*_*p*_ and *pp* are two essential parameters [14]. Pulse ablation threshold fluence (*Th*_*p*_) usually decreases with increase of *pp* due to incubation effect [15]. Therefore, *F*_*a*_ is an important parameter to form homogenouse morphology of HSFL. *F*_*a*_ should be higher than the ablation threshold (*Th*_*a*_) and lower than the disordering threshold of accumulated fluence [16, 17]. The different laser polarization gives rise to a different morphology of HFSL. In this paper, we employed the linearly polarized and elliptically polarized beams to investigate the polarization dependent morphology of HFSL.

#### 3.1.1 Linearly polarized beam processing

**Figure 1:**
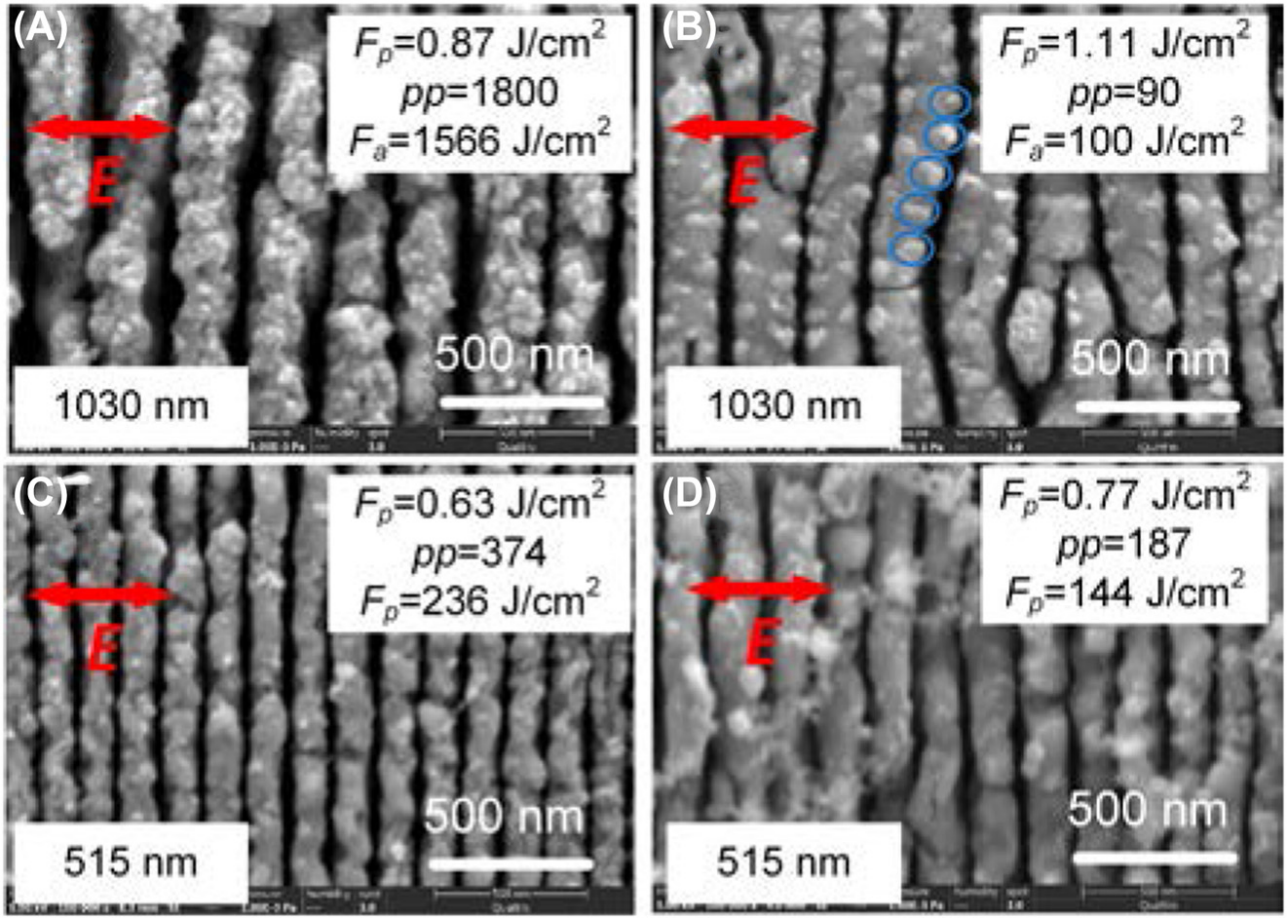
The morphologies of HSFL fabricated by linearly polarized ultrafast laser scanning with a pulse width of 223 fs. (A) *F*_*p*_ = 0.87 J/cm^2^, *pp* = 1800 in 1030 nm. (B) *F*_*p*_ = 1.11 J/cm^2^, *pp* = 90 in 1030 nm. The sites of spallative ablation are indicated with blue circles. (C) *F*_*p*_ = 0.63 J/cm^2^, *pp* = 374 in 515 nm. (D) *F*_*p*_ = 0.63 J/cm^2^, *pp* = 1800 in 515 nm.

Figure 1A–D show SEM images of HSFL formed on ZnO substrates by 1030 nm and 515 ultrafast laser with a pulse width of 223 fs, respectively. The orientation of HSFL was perpendicular to the laser polarization regardless of *F*_*p*_, *pp*, laser wavelength, and scanning direction (Figure S2). For 1030 nm laser, the period of HSFL was evaluated to be ∼262 nm, while ∼136 nm for 515 nm laser. The widths of nanogroove sizes for 1030 nm and 515 nm were ∼69 nm and ∼25 nm, respectively. Considering ZnO is transparent to 515 nm ad 1030 nm lights, the LIPSS formed by both wavelengths is LIPSS-2, so that the period of HSFL can be calculated by: *λ*/2*n*. According to this formula with a refractive index of 1.96 for ZnO, Period_1030,_ and Period_515_ are calculated to be 264 nm and 132 nm, respectively. Both calculated values are very close to the experimental results. Figure 1A shows an over-ablated morphology in which the nanoridges were ablated to form a grain-like structure. In the meanwhile, as seen from the blue circles with much lower *F*_*a*_ induced the periodic nanoablation at both edges of nanoridges. This periodic ablation may be caused by the near-field ablation (spallative ablation) in laser scanning with relatively high *v* [18]. This spallative ablation has a coherent characteristic with a smaller period than HSFL. At the edges of nanogrooves, the laser field induces the enhanced near-field SPP, which leads to periodic melting and ablation of the material. In addition, although the period of HSFL was not affected by *F*_*p*_, *pp,* and laser wavelength, the nanogroove width increases slightly with increasing *pp* due to the metallic transition of ZnO, which has been investigated previously (Figure S3) [9, 19, 20]. This metallic transition of dielectrics supports the SPP generation and fulfills the metallic condition, resulting in the real part of permittivity (*ε*’) of the laser-excited surface smaller than −1. In detail, *ε*’ = *ε*_*c*_ – *ω* ^2^ *_p_*/(*ω* ^2^ + Γ^2^) and *ω* ^2^ *_p_* = e^2^ *n*_eff_/(*m*_eff_ *ε*_0_), where *ε*_*c*_ is the permittivity of ZnO at the normal state, *ω* is the incident laser frequency, *ω*_*p*_ is the plasma frequency, *n*_eff_ is the effective refractive index, and *m*_eff_ is the effective mass of electron. Thus, the permittivity of ZnO is related to the *n*_eff_, which is determined by the laser-excited electron density [21, 22]. Consequently, as the pulse overlap increased, the laser-excited electron density increased, leading to slight increase of the HSFL period. We also investigated the HSFL was transited to LSFL by decreasing *pp* to 8 (Figures 4A). The homogenous HSFL was created in a large area of the ZnO substrate at *F*_*p*_ = 0.95 J/cm^2^, *pp* = 180 (Figure S4B), which would be used for SERS substrate with gold coating.

#### 3.1.2 Elliptically polarized beam processing

**Figure 2:**
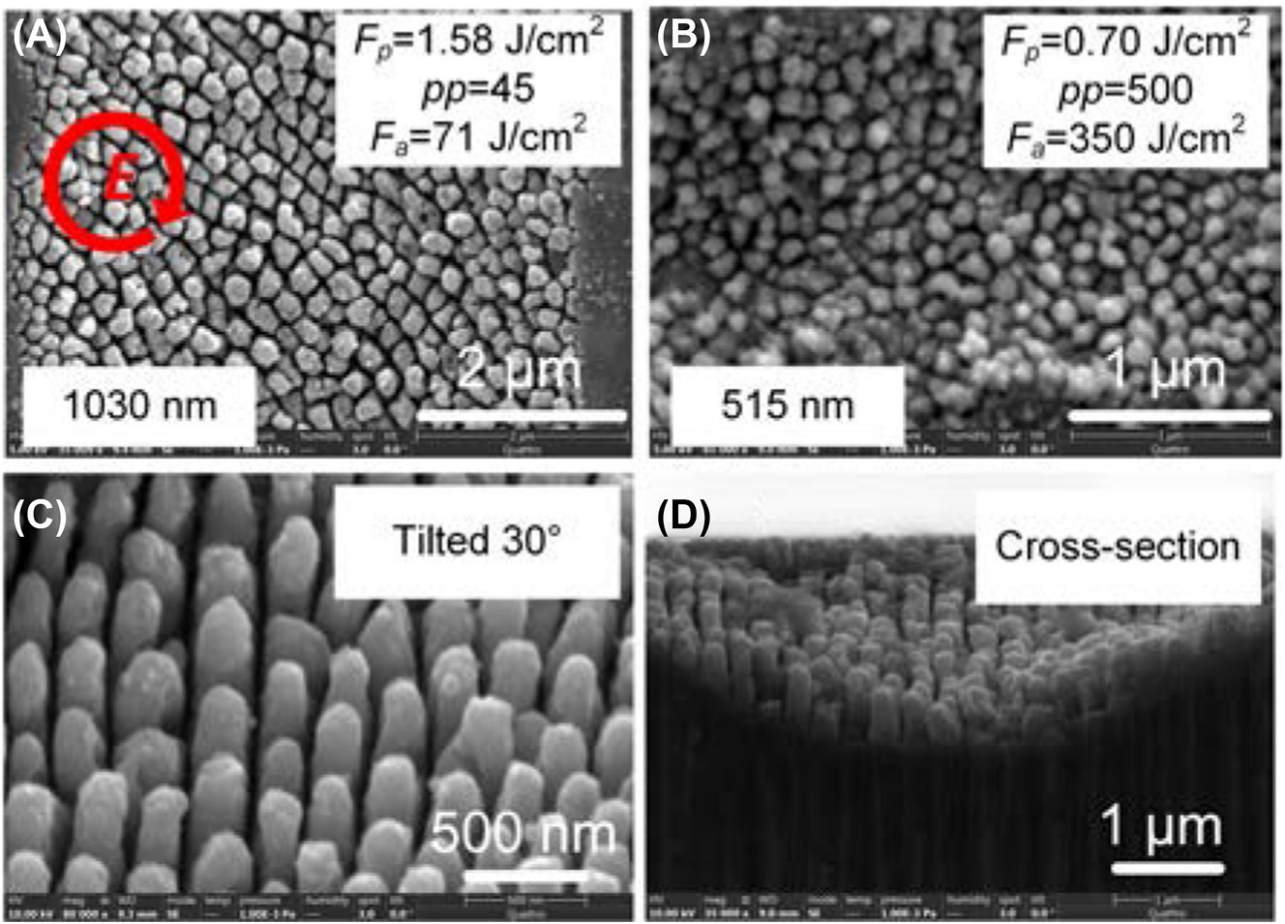
The morphologies of HSFL fabricated by elliptically polarized ultrafast laser scanning with a pulse width of 223 fs. (A) *F*_*s*_ = 1.58 J/cm^2^, *pp* = 45 for 1030 nm. (B) *F*_*s*_ = 0.7 J/cm^2^, *pp* = 500 for 515 nm. (C) 30° tilted and (D) cross sectional images of (A).

Different from the nanostrip structures fabricated by the linearly polarized beam, the nanopillar array was fabricated on the ZnO surface using an elliptically polarized beam for both 1030 nm (Figure 2A) and 515 nm (Figure 2B). We created histograms of the nanopillar width and the gap of nanopillars. In Figure S5A and B, the average size of nanopillar and the gap distance fabricated by 515 nm were measured to be ∼97 nm and ∼24 nm (<*λ*/20), respectively. From the tilted (Figure 2C) and cross-sectional (Figure 2D) SEM images, the height of nanopillars was estimated to be ∼350 nm.

**Figure 3:**
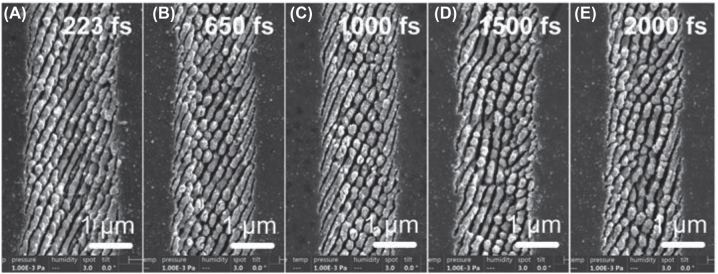
HSFL fabricated using circularly polarized laser beam with pulse duration from 223 fs to 2000 fs. The processing parameters: *F*_*p*_ = 1.26 J/cm^2^, *pp* = 37.

For the elliptical polarization, the shape of nanopillar is related to the ellipticity (Ellipt) of laser beam polarization. Ellipt = (*a* − *b*)/*a*, where *a* is the length of long axis and *b* is the length of short axis of elliptically polarized beam. In Figure S6, we compared the morphologies of HSFL for Ellipt = 4/7, 1/3, and 0. At Ellipt = 4/7, nanostrips were fabricated on the surface, whose direction was perpendicular to the long axis. The nanopillar appeared at Ellipt = 1/3 and became homogenous array at Ellipt = 0. Meanwhile, the self-organization of material also contributed to the formation of nanopillar array due to the SPP coupling between adjacent nanopillars [23] and Coulomb explosion [24], which indicated that the pulse duration should be an important parameter for nanopillar array formation. As shown in Figure 3, we investigated the nanopilliar array using the circular polarization with different pulse durations (223 fs–2000 fs). Because the time of SPP was extended by longer pulse duration, the nanopillar was more obvious at 2000 fs, while at short pulse duration (223 fs), the structure became nanostripe.

### 3.2 *λ*/20 or smaller nanostructure formation

#### 3.2.1 Nanogrooves

**Figure 4:**
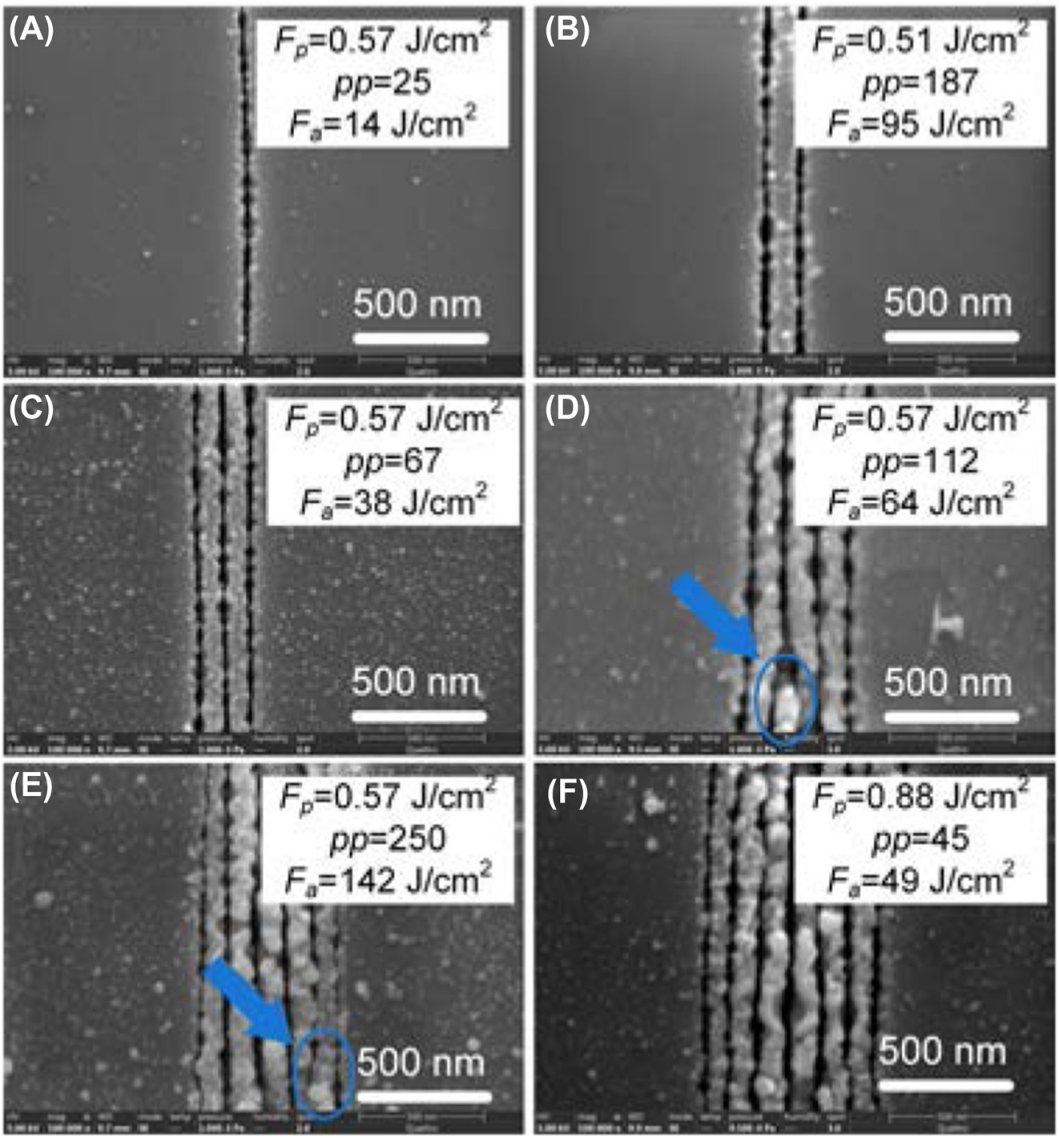
Single and multiple nanogroove fabricated by 515 nm laser scanning. (A) Single nanogroove. (B) Two nanogrooves. (C) Three nanogrooves. (D) Four nanogrooves. (E) Six nanogrooves. (F) Seven nanogrooves.

As explained in Section 3.3.1, the width of nanogroove is proportional to the laser wavelength. To obtain the narrower nanogroove, we employed 515 nm laser. Since the ZnO is transparent material at 515 nm, the laser processing of ZnO should be also based on multiphoton absorption. If the spatial energy distribution of laser with linear polarization has a Gaussian profile, number of nanogrooves created can be controlled by adjusting *F*_*a*_. As shown in Figure 4, at *F*_*p*_ = 0.57 J/cm^2^, *pp* = 25, a single nanogroove was fabricated on the surface with an average width of ∼20 nm (*λ*/25). The multiple nanogrooves with controlled numbers were created by increasing *F*_*a*_ (increasing *F*_*p*_, *pp,* or both). Two nanogrooves was fabricated at *F*_*p*_ = 0.51 J/cm^2^, *pp* = 187; three nanogrooves at *F*_*p*_ = 0.57 J/cm^2^, *pp* = 67; four nanogrooves at *F*_*p*_ = 0.57 J/cm^2^, *pp* = 117; six nanogrooves at *F*_*p*_ = 0.57 J/cm^2^, *pp* = 250; and seven nanogrooves at *F*_*p*_ = 0.88 J/cm^2^, *pp* = 45. However, we found that for an even number of nanogrooves (Figure 4D and E), the defects (pointed by blue arrows) were generated in the nanogrooves occasionally, which may result from the centrosymmetric distribution of Gaussian spot. So the modulated field with an even number of maximum enhancement is unstable and an odd number of nanogroove is preferable to create finer nanogrooves.

#### 3.2.2 Nanocavities

**Figure 5:**
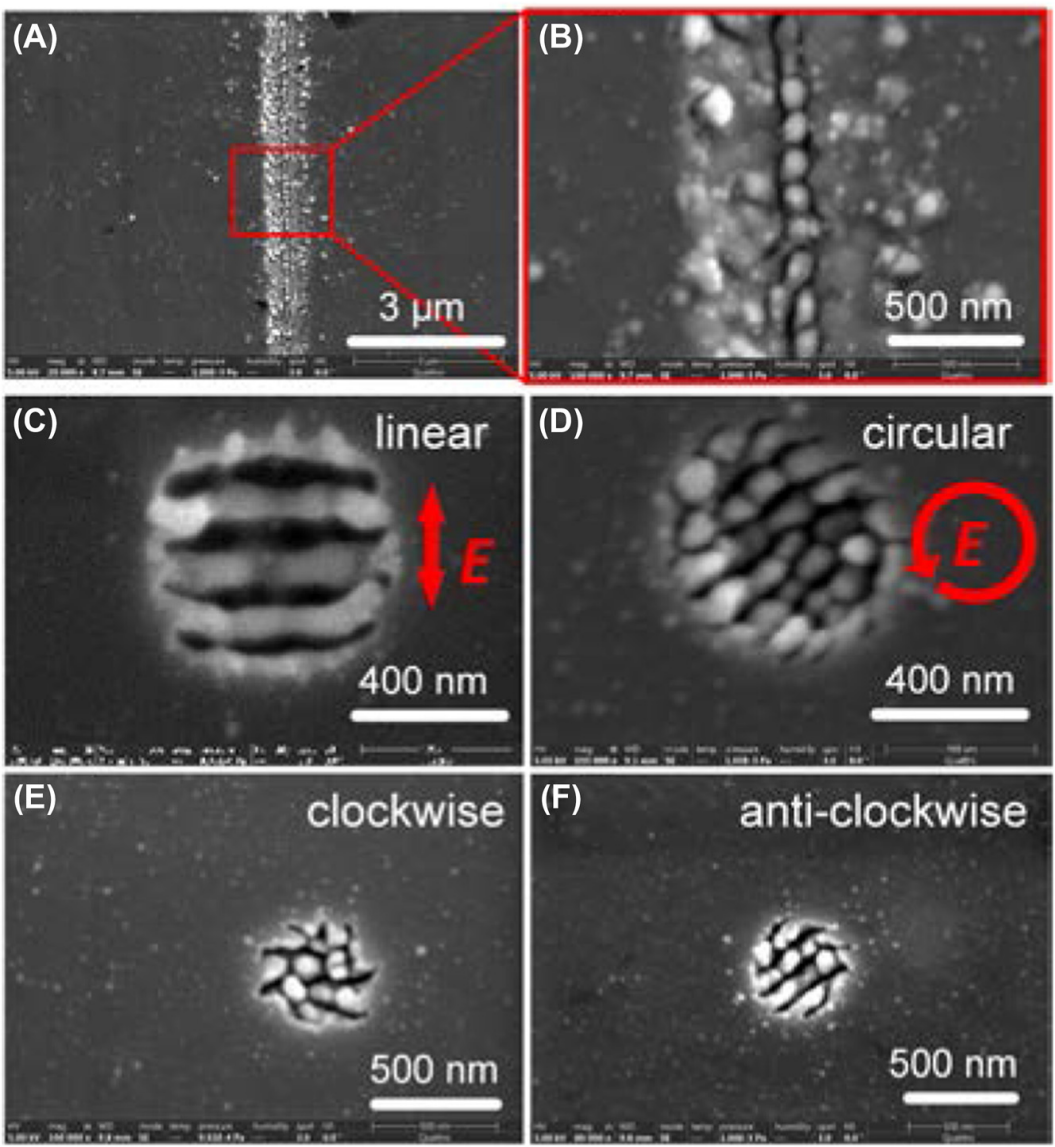
Nanopillar array fabricated by 515 nm laser with circular polarization. (A) and (B) A string of nanopillars in a single line formed by 515 nm circularly polarized laser scanning. The processing parameters: *F*_*p*_ = 1.01 J/cm^2^, *pp* = 9. (C) Nanostrip and (D) nanopillar were created by linear and circular polarizations, respectively. (E) Clockwise and (F) anti-clockwise nanopillar array fabricated by left- and right-hand rotated polarizations, respectively.

With the circular polarization, a string of nanopillar in a single line was created in the center of laser scanned region, as shown in Figure 5A and B. On both sides of nanopillar string, the substrate was melted and ablated because the *F*_*a*_ did not surpass the formation threshold of nanopillar. The string of nanopillar in a double line was also fabricated by increasing the *pp* from 9 to 37, as shown in Figure S7A. To further investigate and compare the HSFL fabricated by linear and circular polarization, the ZnO substrate was irradiated by a focused laser beam at the same location with 20 pulses. As shown in Figure 5C and D, the nanostrips were created by the linear polarization as we illustrated previously, while the nanopillar array was fabricated by the circular polarization. However, we found that the nanopillar array was vortical and the center of the vortex was at the laser spot center. The direction of vortex was determined by the rotation direction of polarization: the left-hand rotated polarization resulted in the clockwise nanopillar array, while the right-hand rotated polarization contributed to the anti-clockwise nanopillar array. This rotated array suggested us to decrease *F*_*p*_ close to the *Th*_*p*_ to inspect what structure would be formed. To this end, we adopted an objective lens with a high numerical aperture (NA = 0.55) to get the laser spot of 1.87 μm in diameter.

**Figure 6:**
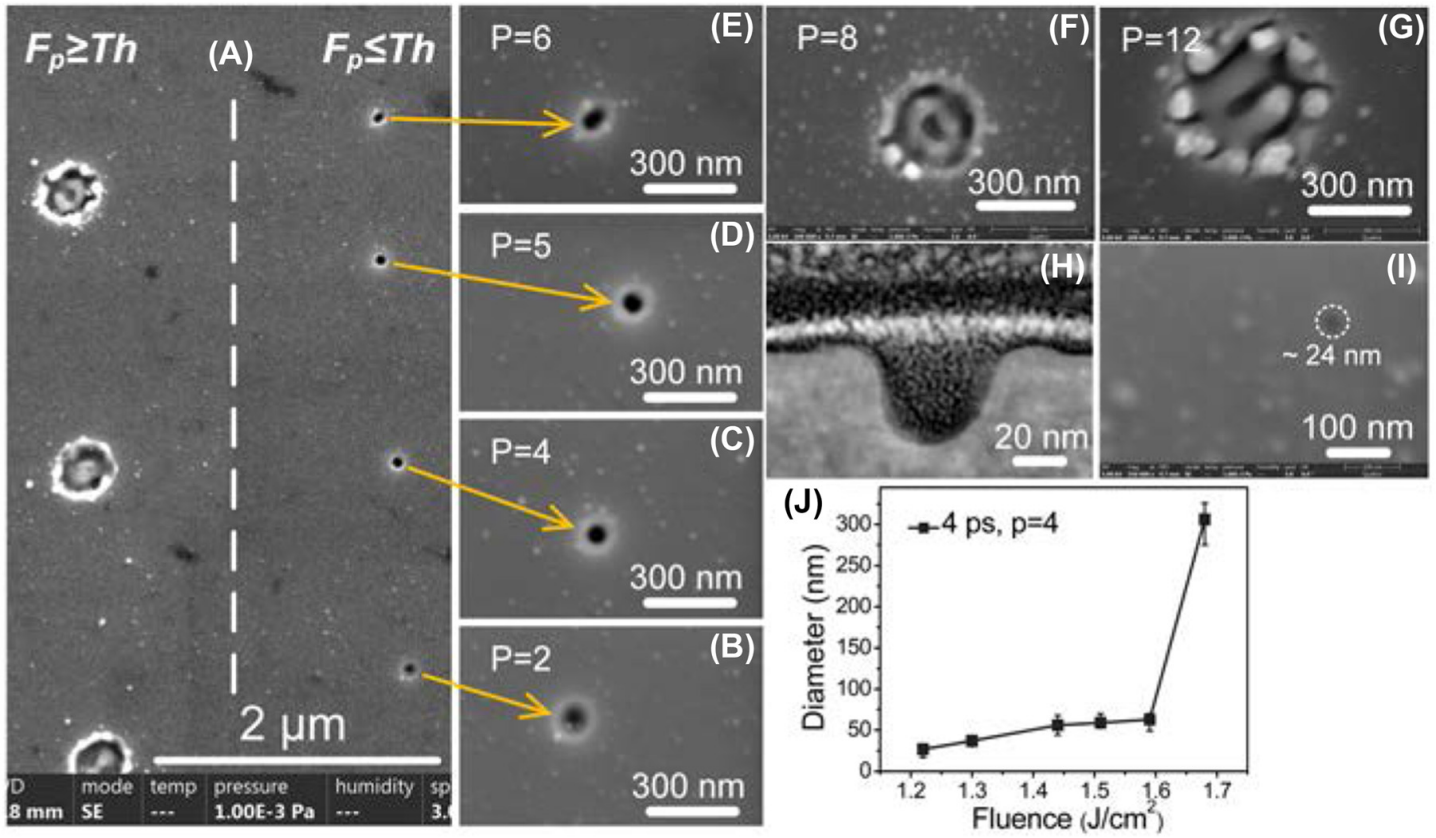
λ/20 single nanocavity fabricated by modulated laser field. (A) Craters and nanocavities fabricated by modulated field by laser processing. *Fp* for crater formation was 1.68 J/cm^2^, while for cavity, 1.59 J/cm^2^. The ablation threshold was around 1.62 J/cm^2^. (B)–(G) The high magnification images of nanocavities fabricated with pulse number of 2–12 at *Fp* of 1.59 J/cm^2^. (H) The cross-section of nanocavity. (I) The minimum nanocavity with a diameter of ∼24 nm. The *Fp* was 1.22 J/cm^2^, and pulse number was 2. (J) The diameters of nanocavities at different fluences.

In laser processing, when *F*_*p*_ is higher than the *Th*_*p*_, the materials start to be ablated. We adjusted *F*_*p*_ close to the *Th*_*p*_ for low pulse number to irradiate ZnO substrate by circularly polarized beam. Interestingly, we found that when *F*_*p*_ was slightly lower than *Th*_*p*_, a single nanocavity was created on ZnO. A typical diameter of the created nanocavity was ∼50 nm (Figure 6 right). However, if the *F*_*p*_ was higher than *Th*_*p*_, large crater was created with a diameter > 300 nm (Figure 6 left). As the SPP induced by the incident laser beam was interfered with the laser field to periodically enhance the field, the modulated field (*F*_*en*_) which is larger than *Th*_*p*_ determines the ablation dimension on the substrate. Therefore, to create nanocavity, multiple pulse irradiation is necessary and *F*_*p*_ should be slightly lower than *Th*_*p*_ *.* Due to the incubation effect, the nanocavity diameter increased from ∼36 nm to ∼60 nm as the pulse number increased from 2 to 6, as shown in Figure 6B–E. The crater was generated at pulse number of 8 and further increase of the pulse number to 12 created a nanopillar array because *F*_*p*_ exceeds *Th*_*p*_ due to the incubation effect. The *F*_*a*_ should be also lower than the LIPSS formation threshold, above which nanopillars were created (Figure S7B). Figure 6H shows a cross-section of a typical nanocavity, whose width and depth are estimated to be ∼44.4 nm (FWHM) and ∼44.2 nm, respectively. The minimum diameter we achieved by this simple approach with careful control of laser irradiation condition was ∼24 nm, which was only *λ*/22 (as shown in Figure 6I). It is worth mentioning that the nanocavity is easier to be fabricated using ps pulse duration than using fs (in Figure 6, a 4 ps pulse duration was employed). Although the SPP lifetime is independent of the excitation pulse duration, the superiority of ps pulse duration for the nanocavity formation may be attributed to the increased coupling efficiency of SPP and the subsequent laser pulse for self-organization of the nanocavity [25]. The matter reorganization as long as 1 s is much longer than the pulse to pulse interval we employed in our experiments (∼100 ms). Additionally, although we did not present in this paper, the nanocavity cannot be obtained when using the linear polarization, because the propagation of SPP induced by linear polarization is limited to a certain direction, resulting in a rod-shaped void with a large size, which has been demonstrated in previous works [26, 27]. Figure 6J shows dependence of nanocavity diameters on *F*_*p*_, indicating slight increase of the diameter as increase of *F*_*p*_ and formation of the crater at *F*_*p*_ above 1.7 J/cm^2^.

**Figure 7:**
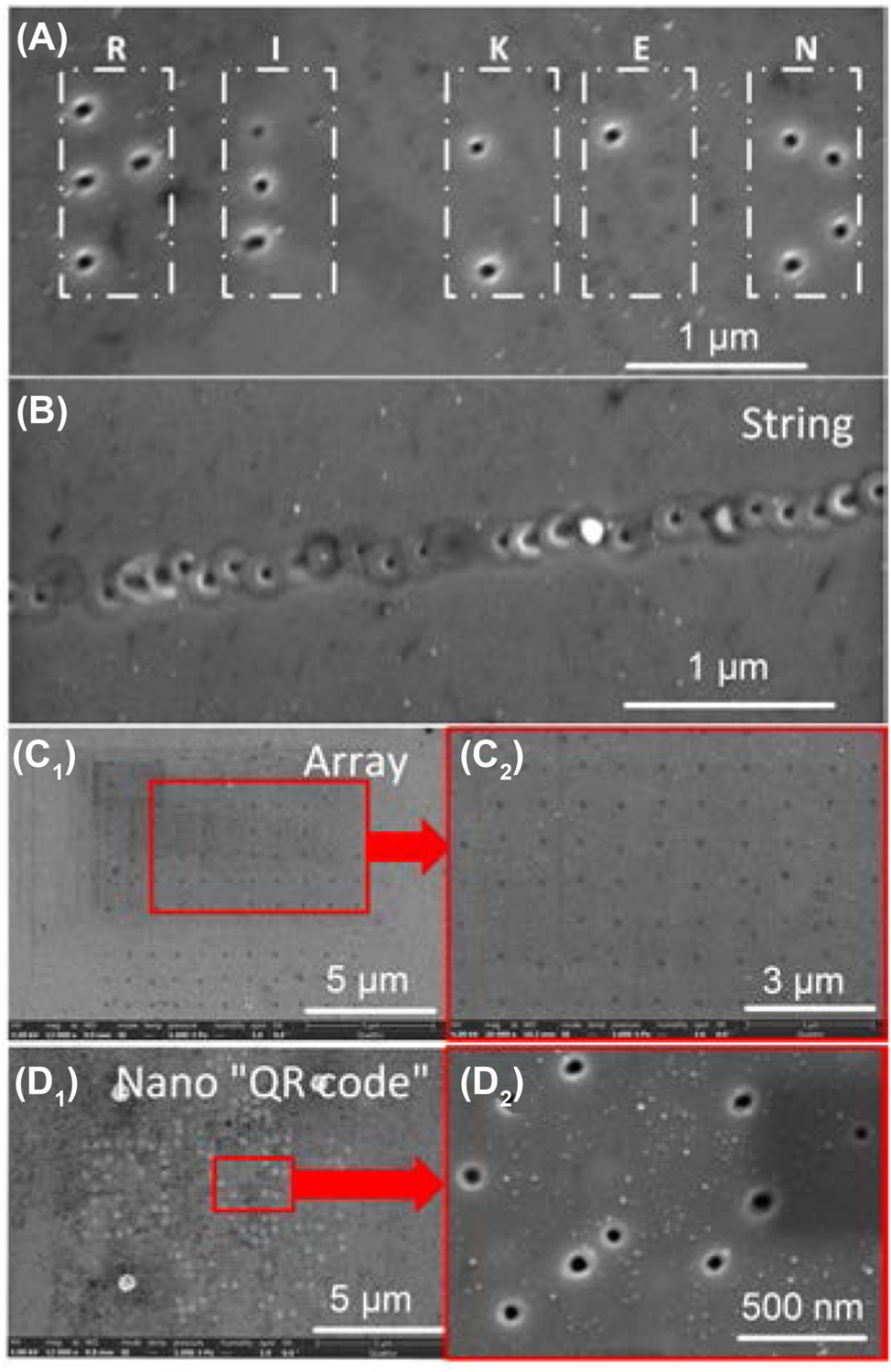
Specific patterns composed of nanocavities fabricated by laser processing. (A) Braille of “RIKEN” composed of nanocavities. (B) A string of nanocavity fabricated by laser scanning. (C_1_ – C_2_) 11 × 11 nanocavity array. The diameter of nanocavits was ∼40 nm. (D_1_ – D_2_) Nano “QR code” generated on ZnO substrate using craters and nanocavities. *F*_*p*_ for crater formation was 1.68 J/cm^2^ with pulse number of 20, while while for cavity, 1.59 J/cm^2^ with pulse number of 4.

Based on this simple approach, several specific patterns composed of nanocavities were fabricated. Figure 7A shows the nanobraille of “RIKEN”, in which the distance between adjacent nanocavities was 300 nm. A string of nanocavity and a 11 × 11 nanocavity array in a 10 × 10 μm^2^ area were fabricated by laser scanning, as shown in Figure 7B and C, respectively. Furthermore, Figure 7D shows a nano “QR code” pattern created in 10 × 10 μm^2^ area. The alignment markers of “QR code” were created by craters and other codes were created by nanocavities. Although the patterns we presented here do not provide specific applications, these results verify ability of flexible patterning of complicated nanostructures by this versatile method for the applications to structural coloring and sensing.

### 3.3 SERS applications

SERS is a spectral technique which may be caused by the near-field optical field effect on noble metal nanomaterials [28, 29]. The collective oscillation of electrons in noble metal nanomaterials enhances the electric field near the surface, which is termed localized surface plasmon resonances (LSPR) [28, 30]. The enhancement is highly related to the distance to the nanostructure surface: *I*_SERS_ = (1 + *r*/*a*)^−10^, where *I*_SERS_ is SERS intensity, *a* is the radius of curvature of the nanostructure and *r* is the distance between the analyte and the nanostructure surface [31]. Thus, the SERS can be only achieved when the analyte is close to the nanostructure surface. Furthermore, because of the strong coupling of LSPR, the intense electric field is induced in ∼10 nm gap regions between adjacent nanoparticles [32]. On the other hand, in semiconductor materials, such as ZnO, the charge transfer between the semiconductor materials and analyte is prominent, contributing to SERS [29]. The metal/semiconductor hybrid substrate is an attractive candidate for highly sensitive SERS substrate, benefiting from the synergistic effect of LSPR and charge transfer. Additionally, to achieve superior SERS performances, the 3D hybrid structure is preferred because it provides larger area of active sites to enhance the Raman scattering. Meanwhile, the laser processing is a versatile method with advantages of maskless process and cost-effectiveness to texture the target. The period and gap size of nanopillars are easily tuned by laser wavelength with relatively high homogeneity, compared to the self-assemble method [33, 34]. The high yield of periodic nanostructure can be achieved by fast laser scanning even in air, which is more promising than focused ion/electron beam lithography. Deep ultraviolet (DUV) laser writing was demonstrated to fabricate ZnO micro- and nano-structures using colloidal ZnO nanocrystals with the ligands or a sol–gel formulation based on zinc methacrylate precursor [35, 36]. However, since this technique relies on direct interaction of laser beam and the material, the fabrication resolution is determined by the optical diffraction limit. In contrast, the smallest nanocavity shown in this paper was ∼25 nm, which was 1/20 of the laser wavelength due to interaction of SPP and the laser beam.

**Figure 8:**
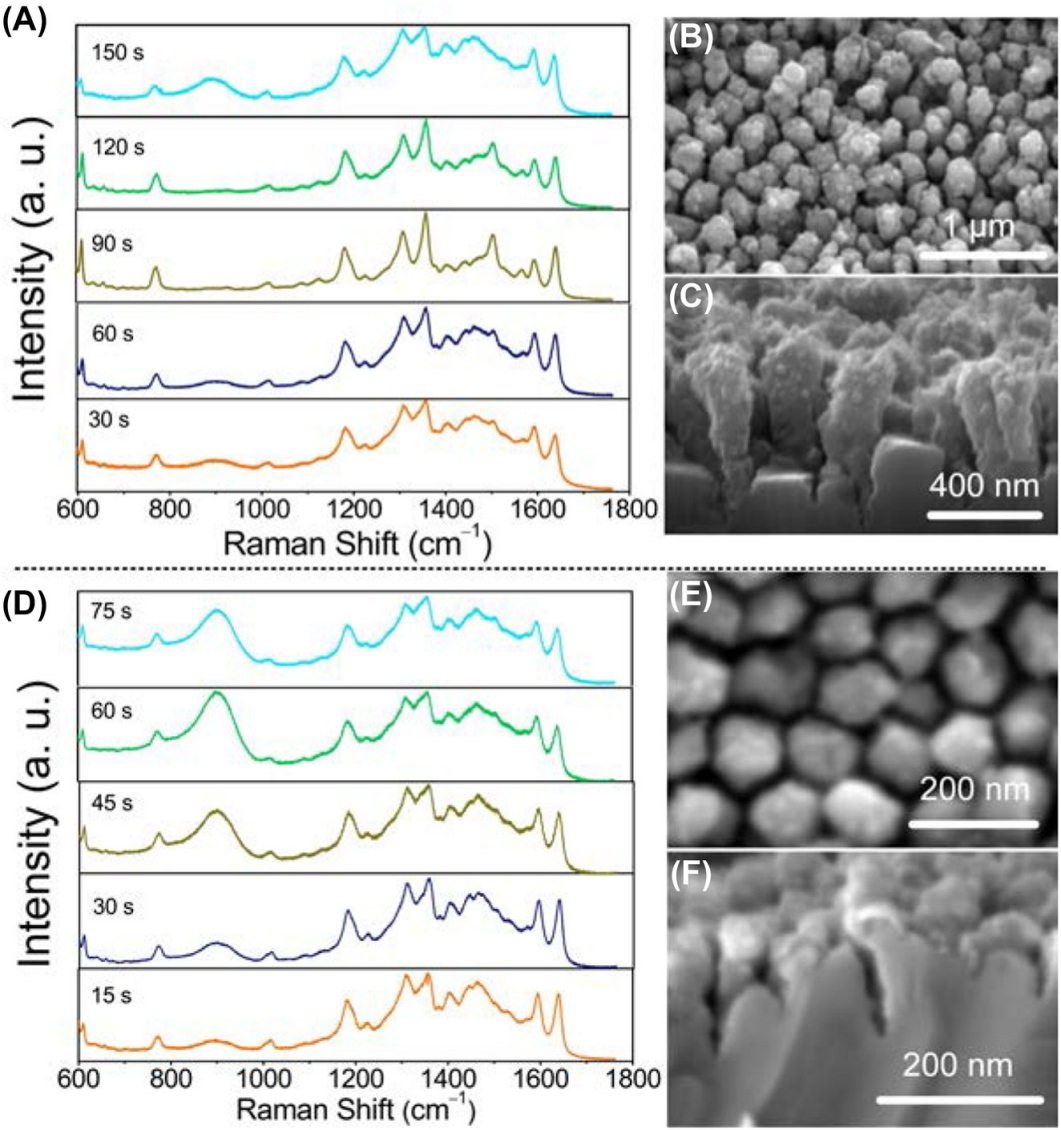
SERS performances on 1030 nm and 515 nm laser fabricated nanopillar arrays. (A) The Raman spectra of rhodamine 6G on gold coated ZnO nanopillar array fabricated by 1030 nm laser with different coating times. The concentration of R6G was 10^−7^ M. (B) Top view and (C) cross section of gold coated zinc nanopillar array. The coating time is 90 s. (D) The Raman spectra of rhodamine 6G on gold coated ZnO nanopillar array fabricated by 515 nm laser with different coating times. The concentration of R6G was 10^−7^ M. (B) Top view and (C) cross section of gold coated zinc nanopillar array. The coating time is 45 s.

To create superior SERS substrates, we coated ZnO nanopillar arrays with gold nanoparticles using an ion sputter. In Figure 8, we evaluated SERS performances of ZnO nanopillar arrays fabricated by 1030 nm and 515 nm laser, both of which were coated with gold nanoparticles for different coating times. Rhodamine 6G (R6G) molecules were used as a test sample. For the 1030 nm laser fabricated ZnO nanopillar array, the highest Raman signals were obtained for the 90 s coating time. After the coating started, the gold nanoparticles gradually deposited on the nanopillar array, and the Raman signals increased with the increase of coating time as the number of gold nanoparticles increases, because the localized electric field increased due to the decreasing of averaged gap distance between each gold nanoparticle. At 90 s, the top and sides of nanopillars were entirely covered by dense gold nanoparticles. Importantly, the gap of adjacent nanopillars coated with gold nanoparticles became sub-20 nm distances as shown in Figure 8B and C, which could induce intense hotspots (Figure S8A). With further coating, the Raman intensity decreased because the gold nanoparticles coated on adjacent nanopillars were connected each other to induce the tunneling effect, resulting in deterioration of LSPR. The Raman intensity was steady after 150 s coating (Figure S8B) since ZnO nanopillars were completely filled by gold nanoparticles, forming gold film. In fact, for the 515 nm laser fabricated ZnO nanopillar array, although it had smaller gaps (∼25 nm), the gold nanoparticles could not be deposited to the side walls of nanopillars since the size of gold nanoparticles was close to the gap size (Figure 8E and F). Consequently, the SERS performance for the 1030 nm laser fabricated ZnO nanopillar array was superior than the 515 nm laser fabricated ZnO nanopillar array (Figure S8D). The SERS enhancement factor (*EF*) of 1030 nm laser fabricated ZnO nanopillar array can be calculated: *EF* = (*I*_SERS_/*I*_OR_)/(*N*_SERS_/*N*_OR_), where *I*_SERS_ and *I*_OR_ are the intensity of SERS and ordinary Raman, respectively; *N*_SERS_ and *N*_OR_ are the numbers of effective excited R6G molecules on SERS substrate and reference substrate (silicon wafer), respectively [37]. Using Raman peaks at 610 cm^−1^ for the calculation, the *EF* was estimated to be 2.28 × 10^7^. However, it is worth to mention that 515 nm laser fabricated ZnO nanopillar array may provide higher *EF* using other gold coating techniques, such as chemical vapor deposition and atomic layer deposition, which will be our future works. Meanwhile, the relative standard deviation (RSD) of the SERS substrate was evaluated to be 10.1% (Figure S8E), based on the SERS measurements at 20 arbitrary locations on the same SERS substrate. The RSD can be improved by using the flat-top laser beam to fabricate the homogeneous ZnO nanopillar array. The reproducibility was further evaluated by comparing the Raman intensities of R6G on two different substrates (Figure S8F). The average deviation of Raman intensity was as small as 6.9%, demonstrating the high reproducibility of this technique (Table S1).

**Figure 9:**
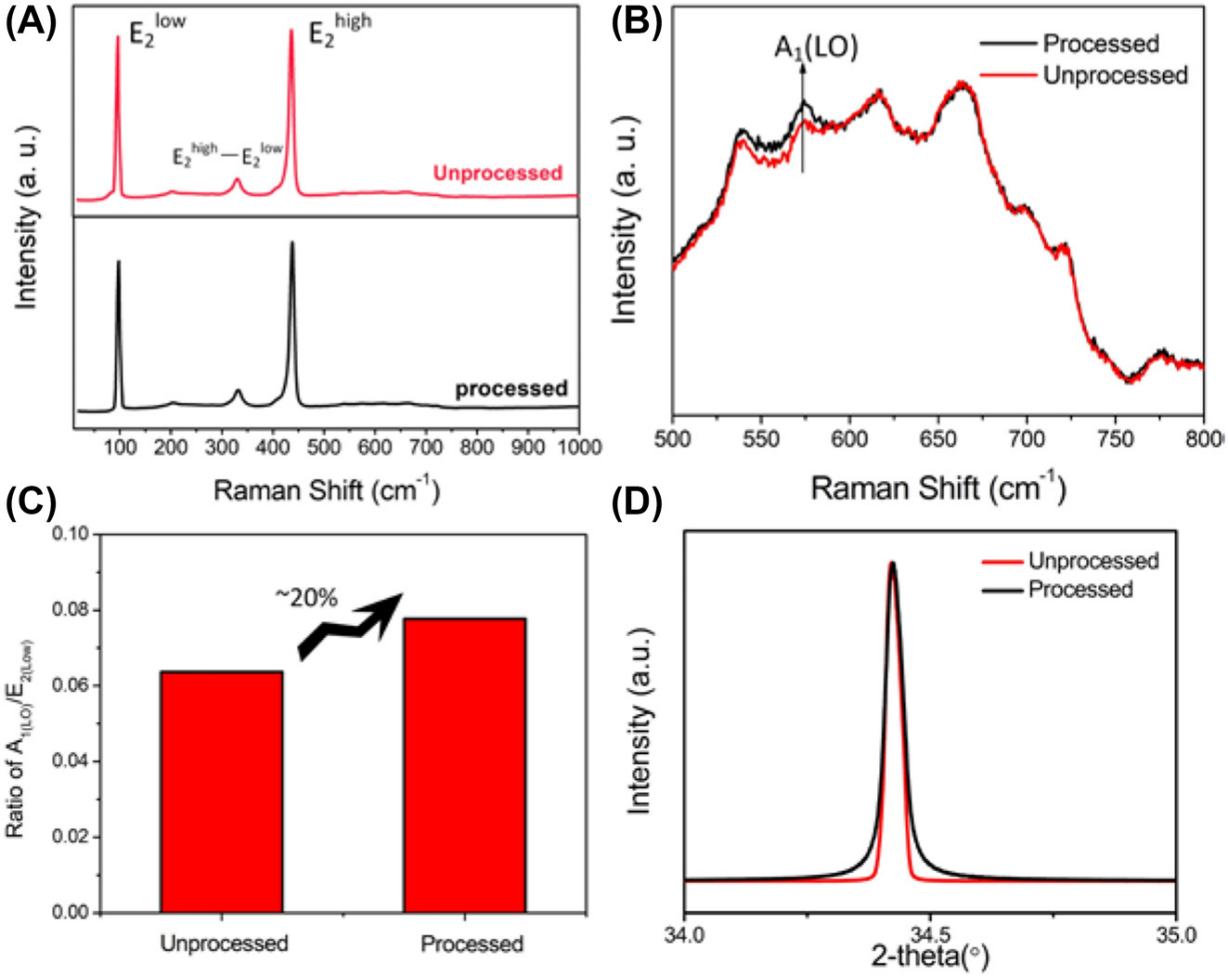
Characterization of nanopillar array. (A) and (B) Raman spectra of original ZnO and laser processed ZnO substrate. (C) The ratio of A_1_(LO)/ $E_{2}^{\text{low}}$ of ZnO substrate. (D) XRD patterns of original ZnO and laser processed ZnO substrate.

It is well known that the ZnO contributes to the SERS by charge transfer, for which the higher concentration of defects ZnO benefits for the achievement of higher *EF* [29, 38], [39], [40]. Figure 9A and B show the Raman spectra of the original ZnO substrate and laser processed substrate (nanopillar array). The main Raman peaks of ZnO were detected, including $E_{2}^{\text{low}}$ at 98 cm^−1^, $E_{2}^{\text{high}}$ at 437 cm^−1^, and $E_{2}^{\text{high}}$ − $E_{2}^{\text{low}}$ at 332 cm^−1^. In Figure 9B, we found the Raman peaks of A_1_(LO) at 576 cm^−1^ increased after the laser processing. The longitudinal optical phonons A_1_(LO) is mainly derived from defects of oxygen vacancy and the Raman intensity ratio of A_1_(LO)/ $E_{2}^{\text{low}}$ increases as the concentration of defects increases [41]. As shown in Figure 9C, this ratio was increased by 20%. The increasing of defects was also verified by XRD. In Figure 9D, in which the diffraction angle of 34.42° was assigned as <002>. Because of the increasing of defects by laser processing, the FWHM of <002> peak became broader compared to original ZnO substrate [42, 43]. The broadening of peak may be also attributed to the decreasing of crystallite size. The crystallite size *t* was determined by Scherrer’s formula: *t* = 0.9*λ*/(*β*cos*θ*), where *β* is the line broadening at full width at half maximum and *θ* is diffraction angle [44, 45]. According to XRD analysis shown in Figure 9D, the crystallite size decreased from 46.03 nm to 19.89 nm.

## 4 Conclusions

In this paper, we presented a simple approach to fabricate *λ*/20 nanostructure on ZnO substrate by direct ultrafast laser processing. At first, we optimized the laser parameters, including *F*_*p*_ *, F*_*a,*_ and *pp*, to fabricate homogenous HSFL. The nanostrip array was obtained by linearly polarized beam. The periods and nanogroove sizes of nanostrip array were 262 nm and 69 nm for the 1030 nm wavelength, and 136 nm and 25 nm for the 515 nm, respectively. The nanopillar array with a height of 350 nm was obtained by circularly polarized beam. The mechanisms of nanostrip and nanopillar array might be attributed to interference of SPP and incident laser beam with self-organization. We investigated the morphologies fabricated at different pulse duration and ellipticity of laser polarization. Furthermore, a single nanogroove and a single nanocavity were created by the linear and circular polarization, respectively. The width of nanogroove and diameter of nanocavity were smaller than *λ*/20 (∼20 nm). Several specific patterns composed of nanocavities were created, including nanobraille of “RIKEN”, a string of nanocavity, nanocavity array, and nano “QR code”. The ZnO substrate with homogenous HSFL was coated by gold nanoparticles for SERS application. The SERS performances were evaluated by R6G, showing an *EF* of 2.28 × 10^7^. The enhancement of Raman scattering was attributed to LSPR based on gold nanoparticles and the charge transfer of ZnO nanopillars. We also verified the charge transfer could be promoted by laser processing due to the increasing of defects in ZnO substrate.

## Supplementary Material

Supplementary Material Details
