# Supplementary material for: λ/20 surface nanostructuring of ZnO by mask-less ultrafast laser processing
Source: Nanophotonics. 2023 Jan 16;12(8):1499–510. doi: 10.1515/nanoph-2022-0657 (PMC11501954; doi:10.1515/nanoph-2022-0657)
Supplement: Supplementary file 1 — Supplementary Material Details [file j_nanoph-2022-0657_suppl.docx]

λ/20 surface nanostructuring of ZnO by mask-less ultrafast laser processing

Shi Bai^1,2^, Zhaoxu Li^2^, Kotaro Obata^1^, Shota Kawabata^1^, Koji Sugioka^1^*

1. Advanced Laser Processing Research Team, RIKEN Center for Advanced Photonics, 2-1 Hirosawa, Wako, Saitama 351-0198, Japan

2. School of Material Science and Engineering, Hebei University of Science and Technology, Shijiazhuang 050018, China


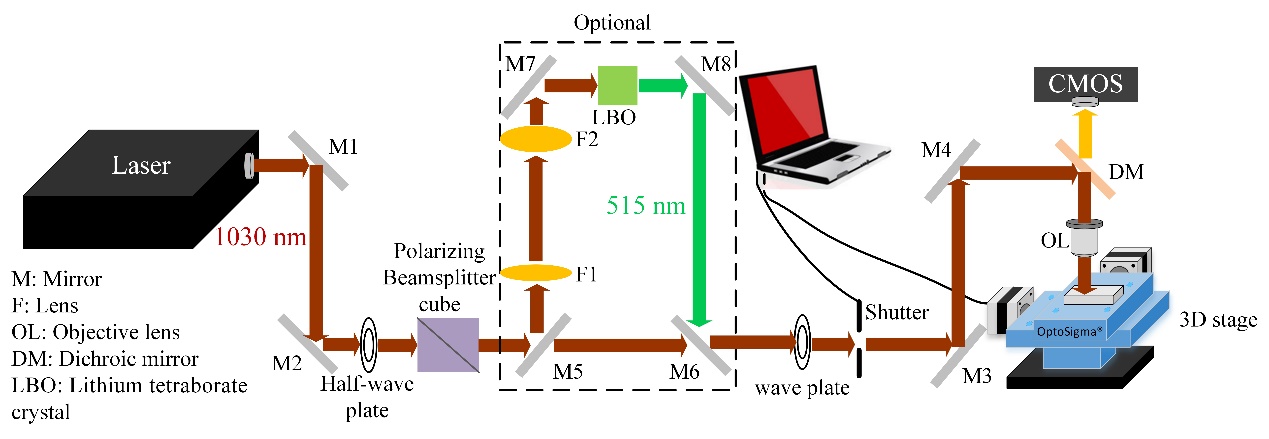


**Fig. S1** Schematic illustration of ultrafast laser fabrication system for surface nanostructuring.


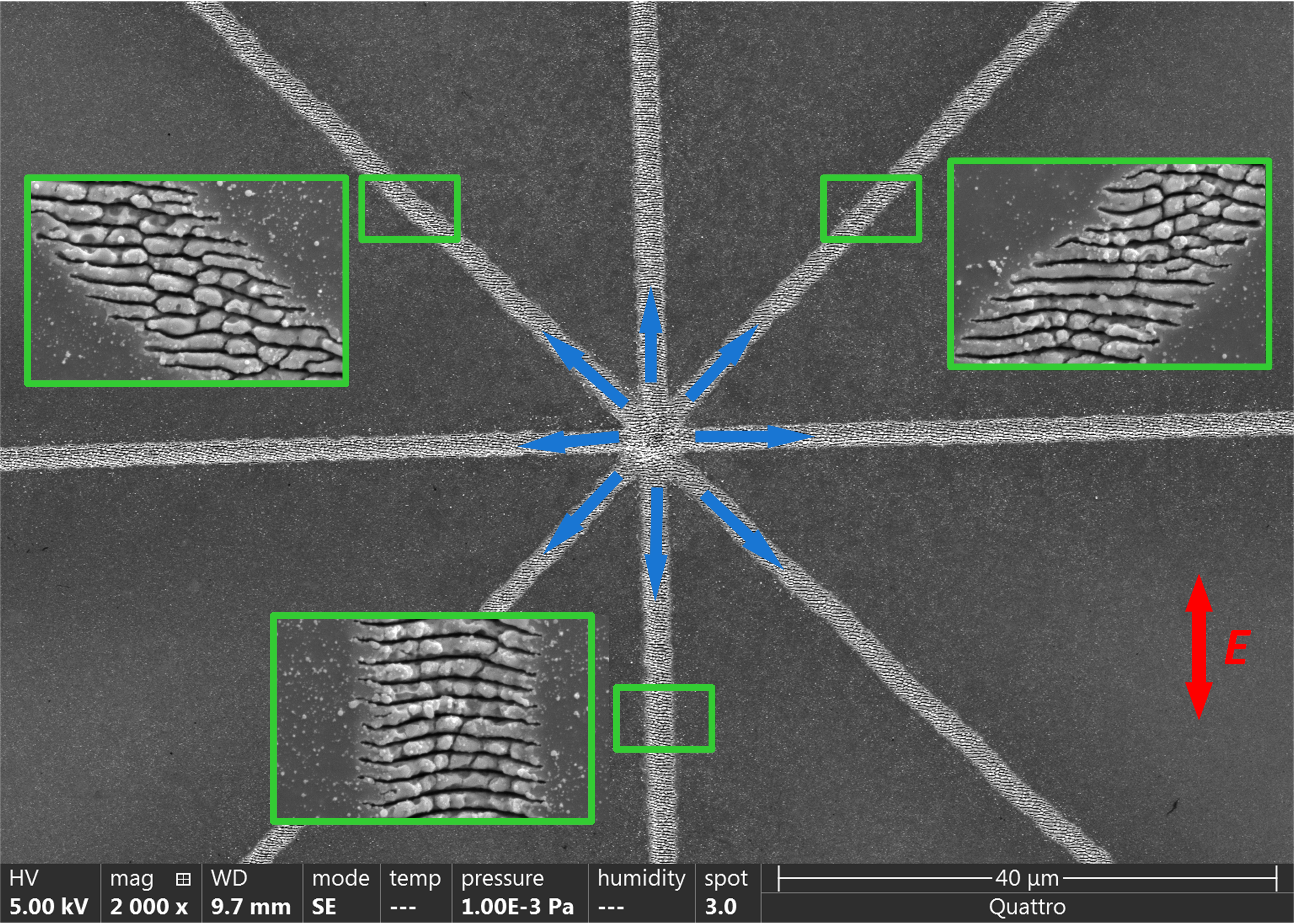


**Fig. S2** HSFL formed on a zinc oxide substrate at different laser scanning directions. The scanning direction was labeled by blue arrow.


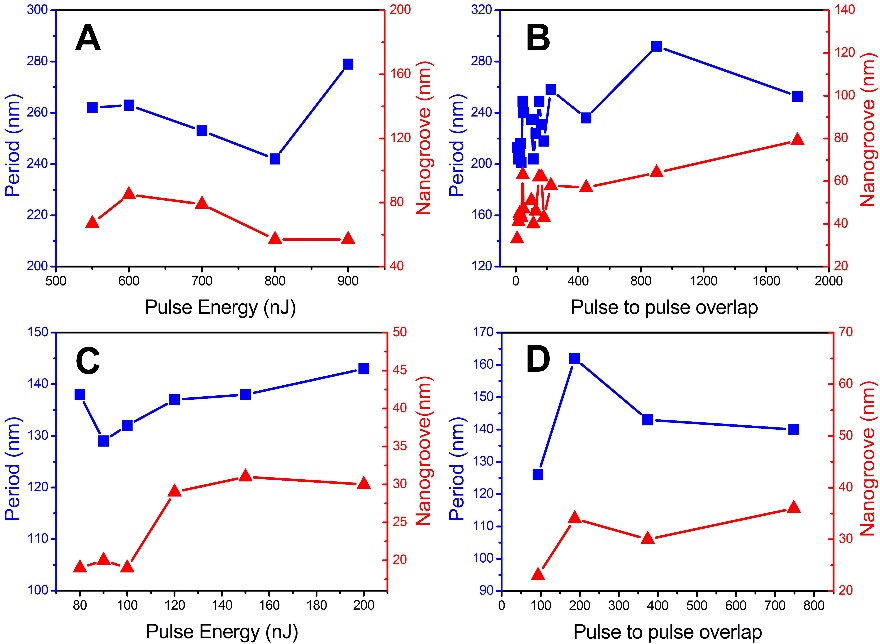


**Fig. S3** Dependence of period and nanogroove width of HSFL on pulse energy and pulse to pulse overlap.


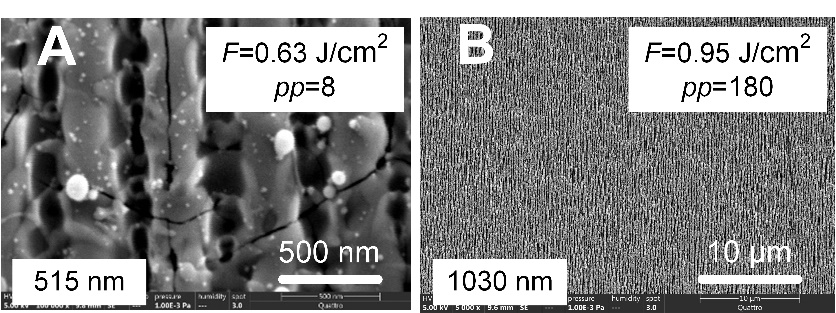


**Fig. S4** (A) LSFL fabricated on zinc oxide substrate. (B) Homogenous HSFL fabricated in large area by laser scanning with optimized processing parameters.

**
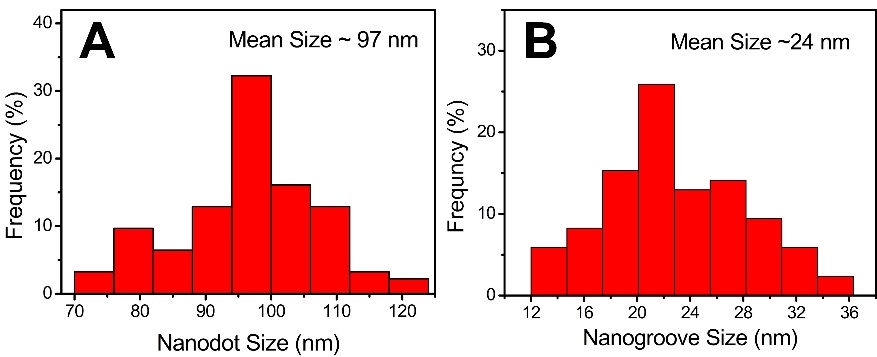
**

**Fig. S5** The size of (A) nanopillar in diameter and (B) gap distance between adjacent nanopillars.


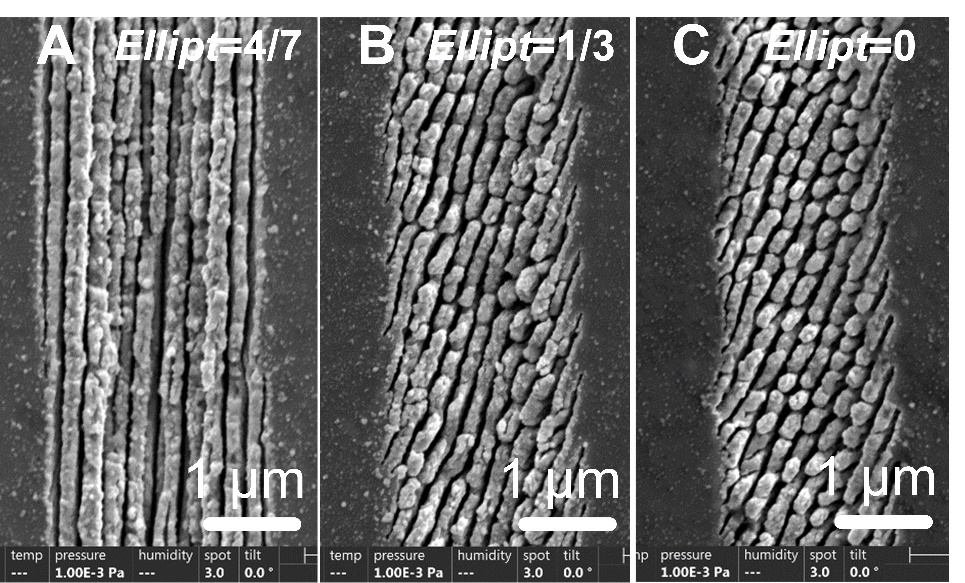


**Fig. S6** HSFL fabricated by elliptically polarized beam at the ellipticity of (A) 4/7, (B) 1/3, and (C) 0.


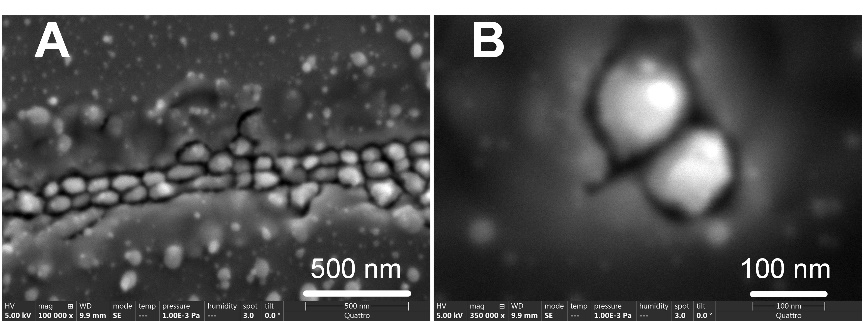


**Fig. S7** (A) A string of nanopillars in a double line by 515 nm circularly polarized laser scanning. The processing parameters: *F_p_*=1.01 J/cm^2^, *pp*=37. (B) Two nanopillars created on a zinc oxide substrate at *F_p_*=1.71 J/cm^2^, pulse number of 12.


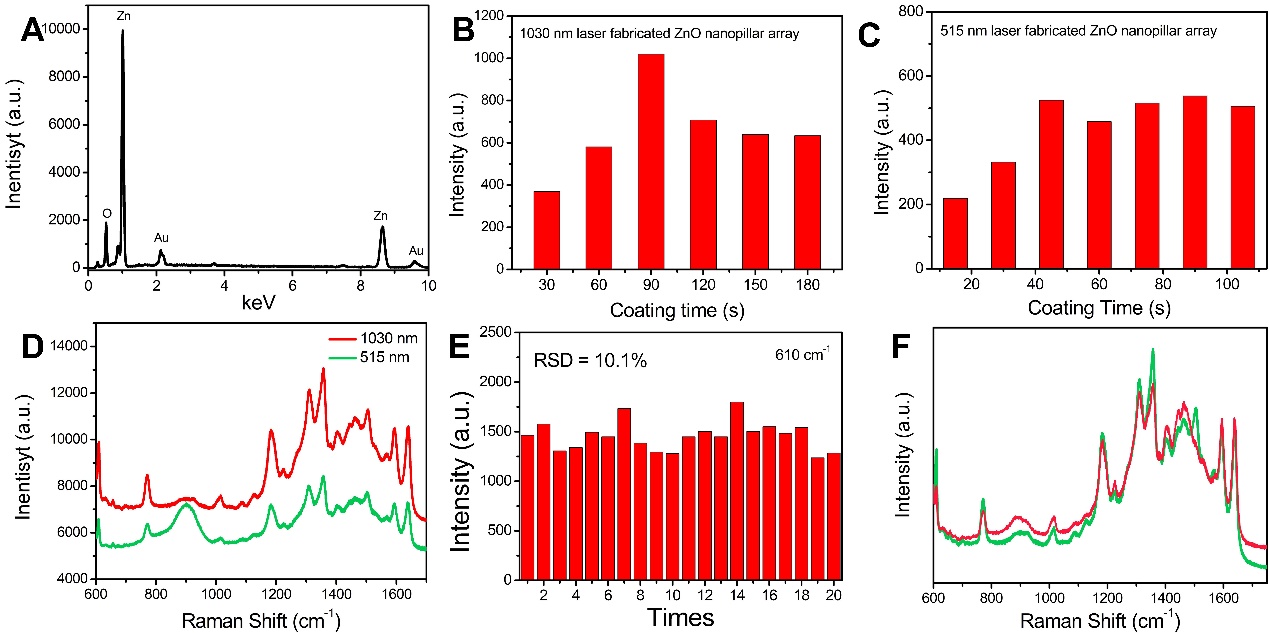


**Fig. S8** (A) EDS spectrum of gold nanoparticle coated zinc oxide nanopillar. (B) Raman intensity of R6G at 610 cm^-1^ on the 1030 nm laser fabricated ZnO nanopillar array with different gold coating time. (C) Raman intensity of R6G at 610 cm^-1^ on the 515 nm laser fabricated ZnO nanopillar array with different gold coating time. (D) Comparison of SERS performances of the 1030 nm and 515 nm laser fabricated ZnO nanopillar array. The gold coating times of 1030 nm and 515 nm laser fabricated ZnO nanopillar arrays were 90 s and 45 s, respectively. (E) Relative standard deviation of Raman intensity of R6G 610 cm^-1^ on the 1030 nm laser fabricated ZnO nanopillar array with 90 s gold coating. (F) Raman intensity of R6G on two different 1030 nm laser fabricated ZnO nanopillar arrays.

**Table S1.** The Raman intensity deviation of R6G on two substrates fabricated by 1030 nm laser at the same conditions.

| Raman peak of R6G (cm^-1^) | Intensity deviation |
| --- | --- |
| 610 | 7% |
| 772 | 3.1% |
| 1183 | 2.6% |
| 1311 | 10.6% |
| 1357 | 15.7% |
| 1595 | 4.9% |
| 1637 | 4.5% |
| Average | **6.9%** |
